# The late preterm IUGR and/or SGA

**DOI:** 10.1186/1824-7288-40-S2-A3

**Published:** 2014-10-09

**Authors:** Enrico Bertino, Luciana Occhi, Paola Di Nicola

**Affiliations:** 1 Neonatal Unit, University of Turin, 10126 Turin, Italy

## 

It is well known that in late preterm infants the mortality and the morbidity are higher than in term neonates. The rate of complications decreases with the progression of gestational age through the late preterm period [1]. Intrauterine growth restriction (IUGR) is one of the cause for late preterm delivery and it occurs more often in late preterm infants than terms ones. Itself constitutes a risk factor for morbidity and mortality [2,3]. IUGR, as well as associated peri-natal morbidities, contributes to increase the risk, in these infants, of postnatal growth impairment, metabolic diseases and poor neuro-developmental outcome [1,4]. Late preterm small for gestational age (SGA) infants were 44 times more likely to die in the first month and 22 times more likely to die in their first year than term adequate for gestational age (AGA) newborns. This increased risk cannot be fully explained by an increasing prevalence of lethal congenital conditions among SGA late preterm newborns [5].

The ability to recognize abnormal growth at birth and or a intrauterine malnutrition is of great importance for the care and the prognosis of these neonates. Neonatal anthropometric charts are commonly used for the diagnosis at birth of SGA newborns [6]. The terms SGA and IUGR are often used as synonyms, however they reflect two different concepts. SGA refers to a statistical definition, based on an auxological cross-sectional evaluation (prenatal or neonatal), and denotes a fetus or a neonate whose anthropometric variables (usually weight) are lower than a given threshold value computed on a set of infants having the same gestational age. IUGR instead refers to a clinical and functional condition and denotes fetuses unable to achieve their own growth potential. Such a condition can be assessed by ultrasonography during pregnancy by a longitudinal evaluation of fetal growth rate. The current gold standard in neonatal auxological evaluation is based on informations obtained from both neonatal anthropometric charts and intrauterine growth charts [7].

At present specific growth charts to monitor postnatal growth of late preterm infants are not available. In the next future the late preterm postnatal longitudinal growth standards will be available as a result of “Intergrowth21st Project”.
